# Development of strategies to enhance recordkeeping during intrapartum care in Limpopo province: A Delphi technique

**DOI:** 10.4102/hsag.v29i0.2645

**Published:** 2024-11-27

**Authors:** Phogole C. Maesela, Johanna M. Mathibe-Neke

**Affiliations:** 1 Department of Health Studies, School of Social Sciences, College of Human Sciences, University of South Africa, Pretoria, South Africa; 2 Department of Health Studies, Faculty of Human Sciences, University of South Africa, Pretoria, South Africa

**Keywords:** Delphi technique, intrapartum care, maternity case record, recordkeeping, strategy

## Abstract

**Background:**

South Africa is experiencing increased medico-legal litigations in maternity services arising from poor recordkeeping. To enhance the quality of recordkeeping and reduce the lawsuits, the strategies were developed and validated by maternal healthcare experts.

**Aim:**

The study is aimed to develop and validate strategies for recordkeeping during intrapartum care in Limpopo province.

**Setting:**

The study was conducted in seven hospitals located in Sekhukhune District, Limpopo province.

**Methods:**

The hospitals were purposefully selected to participate in this study. A sequential explanatory mixed-method design was applied. For the quantitative phase, the maternity case records were reviewed for completeness. Statistician assisted in translation of the checklist into statistical package for social science (SPSS) program. The data analysis included descriptive and inferential statistics. The qualitative phase, focus group discussions and in-depth interviews were conducted with midwives and doctors to describe and determine their perceptions and experiences of recordkeeping during intrapartum. A co-coder was engaged in qualitative data analysis. The findings of quantitative and qualitative phases were integrated to develop the strategies for recordkeeping. A two-rounds Delphi technique was employed to validate the strategies by engaging maternal healthcare experts.

**Results:**

Ten (10) strategies to enhance recordkeeping were developed and validated by the experts.

**Conclusion:**

The proposed strategies were operationalised into interventions with the aim to improve recordkeeping.

**Contribution:**

The strategies aim to improve the quality-of-service provision during intrapartum care and reduce or culminate legal claims in Limpopo province.

## Introduction

The guidelines for maternity care in South Africa (Department of Health [DoH] 2016) stipulate that all observations during labour should be recorded on a partograph. An important component of quality assurance is auditing of the maternity case records, based on the developed standards as suggested by DoH (2016). These standards consist of a list of clinical record headings and a description of the variables that should be recorded under each heading. A study was conducted by a researcher in Limpopo province about the factors contributing to perinatal mortality in 2018 which revealed that 75.3% (*n* = 122) of perinatal mortality was related to poor documentation of the maternity case records by healthcare professionals (Maesela 2018).

Recordkeeping is a challenging task for health professionals working in public hospitals because of several issues, including a limited time to complete the records, an increase in patient admissions and a lack of recording materials as stated by Mutshatshi et al. (2018). Mathioudakis et al. (2016) highlighted that poor recordkeeping does not put the patient at the centre of care, but rather increases medicolegal risks and makes it challenging to track clinical care decisions and care objectives. Proper records management is central to the promotion of good governance. A study conducted by Nabwami (2018) found that preserving records was a professional practice that should support the interventions offered to patients. Furthermore, Putul and Mukesh (2015), on reviewing medical records in day-to-day practice, identified that while physicians acknowledge the importance of medical records to both patient care and medical education, there is an increasing recognition that recordkeeping in our modern method of processing medical data is fundamentally compromised.

### Study objectives

The objectives of this study were to explore recordkeeping during intrapartum care, and develop and validate strategies to enhance recordkeeping in maternity case records by midwives and medical practitioners at the selected healthcare facilities in Sekhukhune District, Limpopo province.

## Research methods and design

The researchers applied a mixed-method approach to the study. A sequential explanatory design was used, where quantitative data were collected and analysed, followed by the collection and analysis of qualitative data (Creswell & Creswell 2022). The quantitative phase entailed the review of maternal case records for completeness, the availability of maternal guidelines and non-participant observations. The qualitative phase was characterised by data collection using focus group discussions with midwives and in-depth interviews with medical practitioners, to determine their experiences and perceptions of recording in the maternity case record. The researcher transcribed and interpreted data from the checklists with the assistance of a statistician by use of the statistical package for the social science (SPSS) version 23, to analyse data, applying descriptive and inferential statistics. Qualitative data were coded and categorised into 10 themes and 53 sub-themes. The integration of quantitative and qualitative findings was achieved through merging, interpretation and meta-inference regarding the phenomenon under study. The themes that emerged from the experiences, challenges and recommendations of midwives and medical practitioners that participated in the study, and the findings from the maternity case records review, informed the development of the strategies through a Delphi technique process (Stewart et al. 2017).

### Setting

The study was conducted in selected healthcare facilities in Sekhukhune District, Limpopo province. Two regional and five district hospitals were purposefully selected to participate in the study. The setting for the development and validation of strategies was online.

### Strategy development and validation process

**FIGURE 1:**
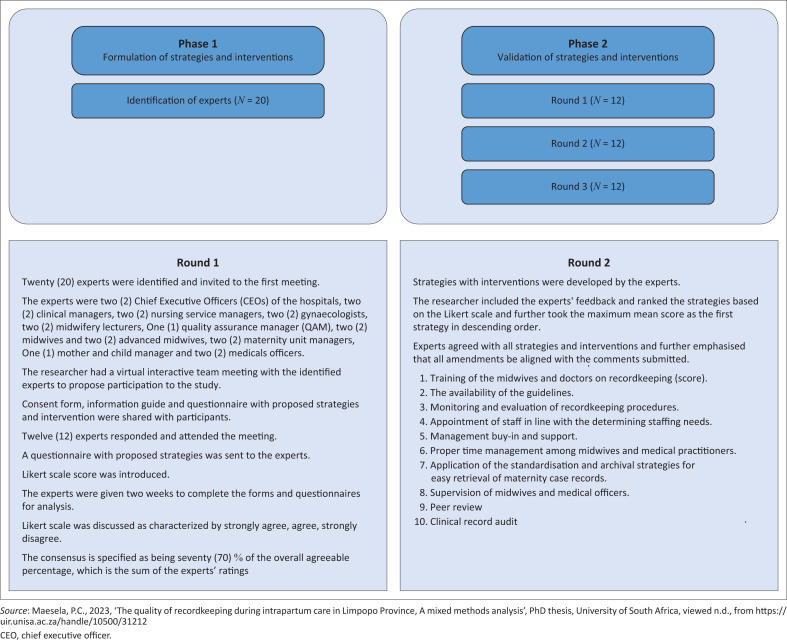
Recordkeeping strategy development process.

The strategy development process refers to a deliberate process whereby the researcher brings together the quantitative and qualitative findings in a study to create a holistic understanding of recordkeeping (Fetters & Molina-Azorin 2017:293). In this study, the researcher formulated the strategies by combining the quantitative results and qualitative findings and validated them by experts through two rounds of a Delphi technique process. An online meeting was held between the researcher and experts to present the study results. An email communication was sent to the experts separately. A Likert scale was used to evaluate the attributes of quality strategies by indicating the level of agreement as strongly agree (4), agree (3), disagree (2) and strongly disagree (1) (Salutini et al. 2020). The interaction between the researcher and experts lasted for 1 h and 30 min, approximately 90 min. Figure 1 illustrates the method followed in the process of strategy development.

### Study population and sampling strategy for the Delphi process

The population for this study were midwives and medical practitioners. The strategies were formulated by the researcher and validated by the experts who comprised of skilled and experienced individuals from various work disciplines who were purposefully selected. McPherson, Reese and Wendler (2018) recommend an 8 to 20 expert panel comprised of participants from different disciplines sampled based on their skills and expertise. The experts included a chief executive officer (CEO) of the hospital, a clinical manager, a nursing service manager, two gynaecologists, a midwifery lecturer, a quality assurance manager (QAM), a midwife and an advanced midwife, a maternity unit manager, a mother and child manager and a medical officer.

The criteria for participating in the Delphi process were that they should be more than 18 years old, willing to participate, had 4 years or more experience working in maternal healthcare facilities.

### Data collection

Twenty experts were recruited to participate in the Delphi process as guided by Maree (2016). Of the 20 experts invited, 12 responded and voluntarily agreed to participate in the validation process. The aim of the validation of strategies was to ensure the reliability and validity of the strategies.

A letter of invitation outlining the purpose of participation, the consent form, the integrated quantitative and qualitative findings, and a questionnaire were sent to the experts. Those who consented to participate were invited to a Microsoft Team Meeting for the researcher to present the integrated study findings. The participants were requested to complete the consent form and return it to the researcher within 2 weeks, to ensure voluntary participation.

The questionnaire consisted of demographic information of the expert and 10 developed strategies, interventions and level of score. The participants had to review the developed strategies, rate each and approve or make suggestions of what should be amended. The experts were given 10 days to finalise and return the questionnaires to the researcher. A courtesy email as a reminder was sent to the participants on day nine with a request to submit the questionnaires to the researcher. The experts who did not respond after 15 days were excluded from further participation. Twelve experts ultimately took part in the second round. The level of agreement was recorded, and the findings were communicated to the participants.

The average time for the meeting with the experts was 90 min. The experts submitted the response to the checklist with the scores and comments for consideration. The experts’ inputs were consolidated into a checklist. Participants were then given the checklists after the amendment of the strategies that included their comments. The checklists were used to assess whether the strategies were clear, consistent and applicable. Data were collected in consecutive rounds one and two until a consensus was reached.

### Data analysis

The scores in the questionnaire were rated using a Likert scale. Descriptive statistics were used to analyse the data and to calculate the percentage and mean score (Polit & Beck 2021). Niederberger and Spranger (2020) highlight that consensus among experts on stated variables should be reached. The percentages were obtained based on the frequency of rating and the level of the experts’ agreement with each strategic intervention. The consensus was specified as being 70% of the overall agreeable percentage, which was the sum of the experts’ ratings for ‘strongly agree’ and ‘agree’ responses.

The responses from round one were analysed using concept analysis (Walker and Avant 2019) to modify the round two questionnaires. The strategies and interventions were grouped and reported on.

### Ethical considerations

Ethical approval was obtained from the University of South Africa (NHREC Registration no.: Rec-240816-052). Permission to conduct the study was sought from the management of the facilities. The ethical principles that were adhered to throughout the study were informed consent, autonomy, confidentiality, justice and voluntary participation. All participants signed consent and confidentiality binding forms. Pseudonyms were used to ensure anonymity. Raw data were kept under lock and key and captured data were password protected.

## Results

The findings of this study are consistent with Lorenzetti et al. (2018) who conducted a systematic review of recordkeeping during an emergency and concluded that medical record documentation is a complex process requiring active support from various stakeholders. Moreover, the implementation of recordkeeping standards can be adapted to the demands of existing workflows and the availability of adequate ongoing training. The study further suggested that audit feedback, reminders, templates and multiple interventions are potentially promising approaches to improve physician documentation. Furthermore, future research is needed to confirm and explore other approaches, including machine learning and other emerging technologies, to enhance documentation in emergency settings.

The researcher achieved an acceptable aggregated response rate with input from the experts in this round. Stewart et al. (2017) highlight that an acceptable consensus should be 70% or more on aggregated summative scores of agree and strongly agree on the Likert scale.

Table 1 reflects the demographic characteristics of the Delphi technique participants.

**TABLE 1:** Delphi technique experts’ demographic characteristics.

Number	Gender	Age (years)	Highest qualification	Position	Years of experience
1	Female	40–49	Bachelor of Medicine and Surgery (MBChB)	CEO	18
2	Male	50–59	MBCHB	Clinical Manager	7
3	Male	50–59	MBBS	Gynaecologist	5
4	Female	40–49	MBCHB	Gynaecologist	9
5	Female	40–49	Degree in Nursing	Nursing Service Manager	22
6	Male	40–49	Master’s degree in Public Health	Quality Assurance	19
7	Female	50–59	Degree in Nursing	Lecturer	6
8	Female	30–39	Degree in Nursing	Unit Manager	12
9	Female	40–49	Master’s degree in Nursing	Mother and Child Health Manager	7
10	Female	50–59	Postgraduate Diploma in Midwifery	Advanced Midwife	20
11	Female	30–39	Diploma in Nursing	Midwife	4
12	Female	30–39	MBCHB	Medical Officer	11

*Source:* Maesela, P.C., 2023, ‘The quality of recordkeeping during intrapartum care in Limpopo Province, A mixed methods analysis’, PhD thesis, University of South Africa, viewed n.d., from https://uir.unisa.ac.za/handle/10500/31212

CEO, chief executive officer.

**FIGURE 2:**
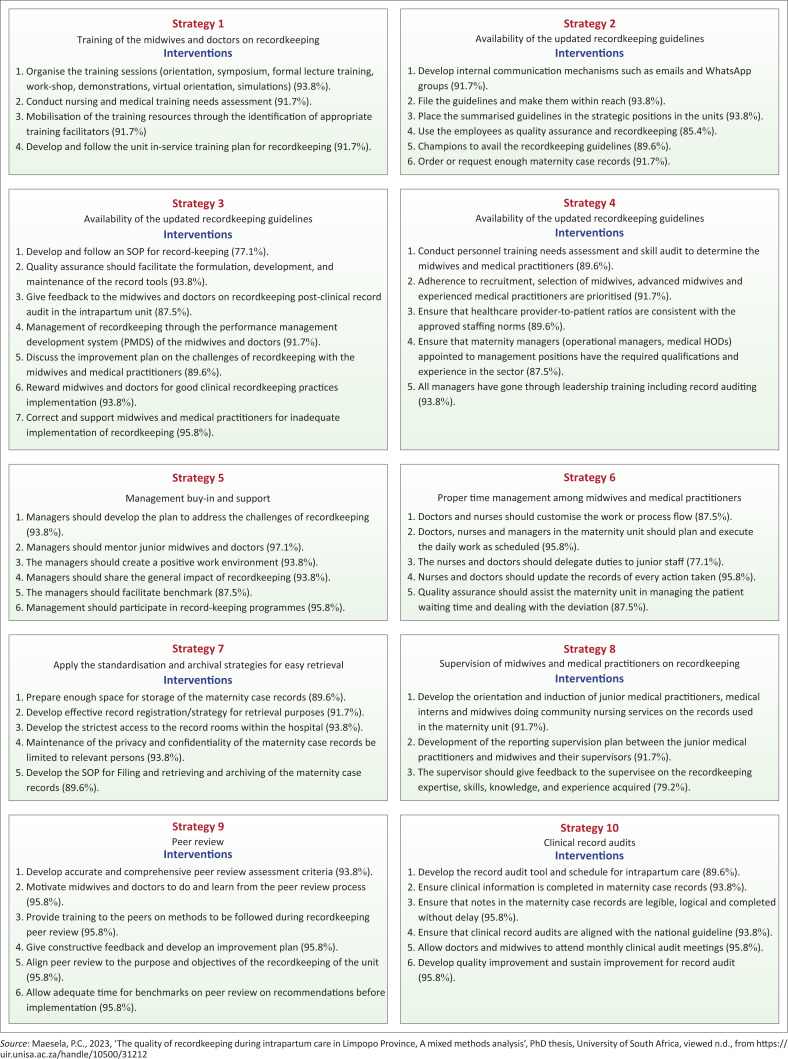
Experts’ scores per strategy.

The Delphi technique process led to the development and validation of 10 strategies and 53 intervention items to improve recordkeeping. The summative scores of these interventions ranged from 77.1% to 97.1%. Figure 2 illustrates the experts’ scores per intervention.

## Discussion of findings

As outlined by Barad (2018), strategies refer to plans to manage specific matters with the aim of achieving objectives or results. In this study, the strategies were developed and validated to be operationalised to address the challenges of recordkeeping during intrapartum care in Limpopo province. Literature was reviewed to support the development of strategies. The literature review was done to complement the content analysis for recordkeeping during intrapartum care. The strategies were operationalised to enhance recordkeeping during intrapartum care in Limpopo province.

### Strategy one

Strategy one was on *training of midwives and doctors*. A comment was made on strategy number one on grammatical errors. The comment made was accepted, and the strategy was amended by the researcher. The consensus was reached that the implementation of the strategy with its intervention by midwives and doctors would bring success in recordkeeping. Improved documentation is the combination of many factors, with the training of the midwives and doctors being used as a vehicle to introduce an improved quality of recordkeeping during intrapartum care. The study conducted in Zimbabwe by Crofts et al. (2015) supported this strategy as it identified that the training of midwives and medical practitioners on recordkeeping is more likely to bring success.

### Strategy two

Strategy two was on the *availability of the guidelines*. The field experts recommended that the management should avail the guidelines in the strategic areas. Consensus was reached by all the experts on this intervention. This study was supported by Stokes et al. (2016) who found that an effective implementation strategy requires flawless guidelines distribution and availability.

### Strategy three

Strategy three was on *monitoring and evaluation of recordkeeping* procedures. Two experts strongly disagreed with the intervention that the quality assurance department should formulate the tools for recordkeeping. The two experts recommended that quality assurance should formulate and review the record audit tools in collaboration with other hospital units. The standard was amended by the researcher and was accepted by all the experts.

### Strategy four

Strategy four reflected on *the appointment of staff* in line with staffing needs. Consensus was achieved on all interventions. According to the study findings, midwives find it challenging to maintain high standards of recordkeeping because of a shortage of midwives in the maternity wards. This notion is supported by Suhaimi, Mulud and Sharoni (2021), in their study on perceptions of midwives on the shortage and retention of staff at public health in Tshwane District, who found it challenging to maintain high standards of recordkeeping throughout intrapartum care because of a shortage of midwives in the maternity wards.

### Strategy five

Strategy five was on *management buy-in and support*. No comments were received to amend the strategy. Macfarlane (2019) indicates that effective clinical governance is important to achieve clear structures and systems, with organisational and clinical administration creating a caring culture, thus it should be the priority for all maternity care.

### Strategy six

Strategy six was on *proper time management* among midwives and medical practitioners. No comments were received to amend the strategy. All the experts agreed on the intervention that both midwives and medical practitioners require time to complete the maternity case records during intrapartum care as participants reported that the completion of the maternity case record is time-consuming and laborious.

### Strategy seven

Strategy seven was on the *application of the standardisation and archival strategies for easy retrieval*. All experts agreed with the intervention. No comments were received to amend the strategy. South Africa (2018) on national guidelines for filing, archiving and disposal of patient records in primary healthcare facilities emphasised that provincial and district offices use this guideline to develop their own provincial or district-specific guideline for filing, archiving and disposal of patient records.

### Strategy eight

Strategy eight addressed the *supervision of midwives and medical officers* during intrapartum care. Ramaboya et al. (2020) conducted a study on managers’ support for the implementation of maternal guidelines, in Limpopo province and found that there is limited support in terms of monitoring and supervision of midwives by managers. Furthermore, the study confirmed that limited supervision and monitoring have a negative impact on the implementation of maternal guidelines.

### Strategy nine

Strategy nine was on *peer review*. The strategy recommended the development of accurate and peer-reviewed assessment criteria. Lalloo, Demou and Macdonald (2015) conducted a study in the United Kingdom, University of Glasgow on the impact of peer review audit on occupational health reports and concluded that quality peer review improves the standard of occupational health reports and is associated with a reduction in customer complaints about reports.

### Strategy ten

Strategy 10 was on *clinical audit*. The experts suggested that the audits be performed in accordance with professional standards. Salem et al. (2015) conducted a study at King Saud University, on medical record audit in clinical nursing units of a tertiary hospital and concluded that a well-planned evaluation of medical records and the related clinical documentation practices allows hospitals and physicians to have an accurate view of their current standing regarding accuracy and compliance for medical recordkeeping.

## Recommendations

The researchers recommend the following:

Policy making:

- that policymakers develop quality improvement plans for in-service training of midwives on recordkeeping
- the quality assurance department to participate in the development of guidelines for recordkeeping
- the appointment of midwives, advanced midwives and medical practitioners to be in line with the facility staffing needs
- the application of the standardisation and archival strategies for easy retrieval of medical records.

Clinical practice:

- adequate management support in guiding the recordkeeping process
- managers to ensure the availability of updated guidelines and maternity case records for recordkeeping
- appropriate time management during healthcare interventions to accommodate recordkeeping
- conduct clinical records audits and undertake peer reviews to enhance recordkeeping as a team approach.

Research:

The feasibility and effectiveness of electronic recordkeeping during intrapartum care as aligned to emerging telemedicine be investigated.

## Conclusion

The two-round Delphi process described in this article was useful in developing and validating strategies to enhance recordkeeping during intrapartum care. The developed and validated strategies and interventions will serve as a guide to assist midwives, medical practitioners and managers in improving recordkeeping during intrapartum care. Furthermore, the strategies and interventions will be a source of reference in the development of guidelines for quality recordkeeping and reduce medical lawsuits emanating from poor recordkeeping. The gap between current and best practices and how to improve recordkeeping need to be explored.

### Limitations

Not all invited experts participated in the study. There were no incentives to reimburse the experts for the equipment which includes data, scanned copies of consent forms and the time they used during the virtual meeting for this study.
